# Correspondence

**Published:** 1912-10

**Authors:** Julius Richter


### CORRESPONDENCE.

Dr. A. L. Benedict,
228 Summer St.

My dear Doctor:—In the last issue of the Buffalo Medical Journal, you ask for information regarding periodically reappearing dermal marks.

I am well acquainted with a young man who has an oblong, vividly red mark, about the size of a five-cent piece on the inner aspect of his knee. This mark promptly makes its appearance in June of every year and just as promptly disappears about the beginning of September. The mark is probably of angiomatous origin and akin to a nævus flammeus or "port-wine" mark.

This young man's mother states: that during her pregnancy with this child, she had an inordinate craving for water-melon, and reasons that the mark on her son, "is the mark of a water-melon seed and therefore only appears during the water-melon season."

Sincerely,

JULIUS RICHTER.

Sept. 12, 1912.

